# A Comparison of Two Operation Methods Revealed the Risk Factors and the Necessity of LN-prRLN Dissection in Papillary Thyroid Carcinoma: A Retrospective Cohort Study in FUSCC

**DOI:** 10.1155/2020/7162793

**Published:** 2020-09-10

**Authors:** Yunjun Wang, Dezhi Wang, Lili Chen, Kai Guo, Tuanqi Sun

**Affiliations:** ^1^Department of Head & Neck Surgery, Fudan University Shanghai Cancer Center, Shanghai 200032, China; ^2^Department of Oncology, Shanghai Medical College, Fudan University, Shanghai 200032, China; ^3^Department of General Surgery, People's Hospital of Tongling, Anhui 244000, China; ^4^Department of Head &Neck Surgery, Renji Hospital, School of Medicine, Shanghai Jiaotong University, Shanghai 200001, China

## Abstract

**Background:**

Although the American Thyroid Association (ATA) guidelines indicate that central lymph nodes posterior to the right recurrent laryngeal nerve (LN-prRLN) should be routinely dissected, pr-RLN dissection is often neglected due to the high risk of injury to the recurrent laryngeal nerve (RLN). The purpose of this study was to investigate the risk factors associated with LN-prRLN metastasis in patients with papillary thyroid carcinoma (PTC) by preoperative examination and the indications for LN-prRLN dissection.

**Methods:**

A total of 1487 consecutive patients with PTC who underwent total thyroidectomy or right lobectomy plus isthmic resection with central LN dissection (CLND) were divided into two groups: patients with LN-prRLN dissection (group A) and patients without LN-prRLN dissection (group B). Clinicopathologic data were reviewed of the patients who were operated on by the same thyroid surgery team in the Department of Head Neck Surgery, Fudan University Shanghai Cancer Center (FUSCC) between August 2011 and May 2019. The relationships of LN-prRLN metastasis with clinicopathologic characteristics were analyzed by univariate and multivariate logistic regression.

**Results:**

The incidence of LN-prRLN metastasis was 34.1% (129/378). Univariate analysis showed that sex (*P* ≤ 0.001), tumor size (*P* ≤ 0.001), extrathyroidal extension (*P*=0.002), concurrent Hashimoto's thyroiditis (*P*=0.009), cLNMa (central lymph nodes anterior to the right recurrent laryngeal nerve) (*P* ≤ 0.001), cLNMa number (*P* ≤ 0.001), and lateral LN metastasis (LLNM) (*P* ≤ 0.001) were significantly associated with LN-prRLN metastasis in PTC. Multivariate logistic regression analysis revealed that tumor size (*P*=0.039), cLNMa (*P*=0.001), and LLNM (*P*=0.025) were independent risk factors for LN-prRLN metastasis in patients with PTC. Although there was no significant difference between the two groups in recurrence, we found that 4 cases relapsed in the LN-prRLN compartment in group B, while none relapsed in group A.

**Conclusion:**

LN-prRLN metastasis is often identified in patients with PTC. Patients with large tumor sizes, cLNMa and LLNM are at a high risk of LN-prRLN metastasis and should be recommended for careful LN-prRLN dissection.

## 1. Introduction

Papillary thyroid cancer (PTC) is the most prevalent endocrine malignancy globally according to the cancer statistics data in China and SEER data (Surveillance, Epidemiology, and End Results 2004–2013) [1, 2]. PTC often shows a better prognosis due to the slow development of characteristics, while lymph node metastasis has been reported to be as high as 20%–90%, especially in the central compartment region [3–5]. Although the American Thyroid Association (ATA) and Chinese Thyroid Guidelines recommend routine (therapeutic and prophylactic) central lymph node dissection (CLND), approximately 10% of patients suffer from recurrence after the initial surgical treatment, and central neck recurrence accounts for 74% of all recurrent cases [6]. This suggests that there may be some problems in this process.

According to ATA guidelines, the central compartment is subdivided into prelaryngeal (Delphian) lymph nodes, pretracheal lymph nodes, and paratracheal lymph nodes. The left recurrent laryngeal nerve (RLN) is located between the esophagus and trachea, but the right nerve ascends through the fat tissue of the right central compartment [7]. Due to anatomical differences, the RLN of the right side is more superficial than that of the left side. Some fat and lymphatic tissues are present posterior to the right RLN. Thus, the right paratracheal lymph node is further classified into two subregions by the right RLN [8]. The nodes located near the posterior side of the right nerve, upper to the esophagus and prevertebral fascia, are called the lymph nodes posterior to the right recurrent laryngeal nerve (LN-prRLN), which were defined as VIb compartments, and lymph nodes anterior to the right recurrent laryngeal nerve were defined as VIa compartments [9]. The reported prevalence of LN-prRLN metastasis varies from 5.76% to 26% [8, 10–14]. In particular, if minor lymph node metastasis (LNM) is located posterior to the right RLN, it is often neglected, and careful dissection is required because of the high likelihood of damage to the RLN. How to reconcile the protection of the RLN while thoroughly dissecting the central lymph nodes in certain situations is an urgent problem that requires exploration.

The purpose of this study was to investigate the frequency and risk factors related to LN-prRLN, evaluate recurrence, and assist surgeons in determining whether to perform selective LN-prRLN dissection in patients with PTC.

## 2. Materials and Methods

### 2.1. Patients

A total of 1487 patients with PTC who underwent thyroidectomy with LND in the Department of Head and Neck Surgery of Fudan University Shanghai Cancer Center (FUSCC) between August 2011 and May 2019 were retrospectively enrolled in this study. Preoperative assessment included ultrasonography (US), computed tomography (CT) scan, chest X-ray, and measurement of thyroglobulin (Tg), thyroid stimulating hormone (TSH), and anti-Tg antibody levels. US was preoperatively performed to assess the lymph node status and confirm no lymph node involvement in any of these patients. These patients underwent surgical treatment by one surgical team. Only newly diagnosed patients were enrolled. Patients with previous thyroid or parathyroid surgery, previous neck surgery, family history of cancer, and history of neck radiation were excluded. The study was approved by the Ethics Committees of Fudan University Shanghai Cancer Center, and all participants gave informed consent.

The clinicopathological characteristics of the different groups were compared, including sex, age, tumor size, location, multifocality, bilateral involvement, extrathyroidal extension, central lymph node metastasis (cLNM), central lymph nodes anterior to the right recurrent laryngeal nerve (cLNMa), and lateral lymph node metastasis (LLNM). Patients with at least one right lobe lesion were classified into the right lobe lesion group. For patients with multifocal tumors, the size and sublocation of the largest tumor were recorded for data analysis. The tumor size was extracted according to the longest diameter of the tumor and recorded in millimeters. In the patients who did not undergo lateral neck dissection, the number of metastatic lateral compartment lymph nodes was considered zero clinically.

### 2.2. Surgical Technique

All patients underwent total thyroidectomy or right lobectomy plus isthmus with routine CLND. LLNM (cN1b) was diagnosed based on preoperative image examination and fine-needle aspiration cytology or intraoperative frozen sectioning and sampling of suspicious lymph nodes, and patients with LLNM underwent lateral neck dissection.

The central compartment was defined according to the ATA's consensus of CLND [7]. The anatomic boundaries of the central compartment lymph node region were defined as follows: the carotid arteries formed the lateral boundaries, the tracheal margins formed the medial boundaries, the hyoid bone formed the superior boundary, the suprasternal fossa formed the inferior boundary, the anterior surface of thyroid gland formed the anterior boundary, and the prevertebral fascia formed the posterior boundary. The patients were divided into the following four groups according to Bae's method [8]: (1) Delphian lymph node; (2) right central lymph node; (3) left central lymph node; and (4) LN-prRLN. The pretracheal and paratracheal lymph nodes were combined into the central lymph node (CLN) group. LN-prRLN was analyzed separately. All lymph nodes were classified by the surgeon during the operation. The presence or absence of LNM was defined according to postoperative pathological reports.

### 2.3. Pathologic Examination

Postoperative histopathologic results were confirmed by 3 pathologists with over 10 years of experience at our institution. All cases were confirmed as PTC using intraoperative frozen paraffin sections and postoperative paraffin sections.

### 2.4. Follow-Up

Follow-up consisted of neck US examination, triiodothyronine (T3), tetraiodothyronine (T4), TSH, and thyroglobulin (Tg) every 3–6 months. If routine tests indicated recurrence, enhanced CT or FNA would be performed to confirm if additional surgery was needed. We considered the patients disease-free when serum Tg levels after injection recombinant human TSH (rhTSH) were 1 ng/ml, neck US was negative, and Tg antibody (TgAb) was undetectable.

### 2.5. Statistical Analysis

All statistical analyses were performed using Stata (version 12; Stata Corp, College Station, TX). Continuous variables were described using the mean ± standard deviation (SD). Data were compared using Student's *t*-test. Categorical variables were described using frequency and percent and the chi-square test, and Fisher's exact test was used as the appropriate method to calculate the difference. Multiple logistic regression analysis was used to assess the statistical significance of the associations between LN-prRLN and clinicopathologic factors. Odds ratios (ORs) with 95% relative confidence intervals were calculated to determine the relevance of all potential predictors. A *P* value< 0.05 was regarded as significant in the included studies.

## 3. Results

### 3.1. Patient Characteristics

Among the 1478 enrolled patients, the mean age at the first diagnosis was 46.14 years (range, 17–71 years). There were 353 men and 1134 women, with a female:male ratio of approximately 3.2 : 1. Overall, a right lobectomy was performed in 1016 (68.33%) patients, and thyroidectomy was performed in 471 (31.67%) patients. The detailed results are summarized in Table 1. In particular, during the mean follow-up period of 36.9 months, recurrence was observed in 2.35% (35/1487) of patients.

Furthermore, these patients were divided into two groups: group A consisted of patients who underwent LN-prRLN dissection, while group B did not.  Table 2 demonstrates the clinicopathological characteristics of the two groups. Group A had a larger tumor size than group B (*P* ≤ 0.001). Concomitant Hashimoto's thyroiditis (co-HT) was found in 140 (37.0%) and 227 (20.47%) patients in these two groups, respectively (*P* ≤ 0.001). cLNMa, including positive and dissected cLNMa numbers (No.), were significantly higher in group A. Likewise, group A patients had, on average, more metastatic node numbers in the lateral neck (*P*=0.022) than group B. The follow-up time was shorter in group A. These results indicate that in recent years, we have tried to resect LN-prRLN conventionally in patients of a younger age, those with a larger tumor size, and those with more lymph node metastasis.

### 3.2. Predictors of LN-prRLN Metastasis

Next, we divided the 378 patients who underwent LN-prRLN dissection into two groups: LN-prRLN with positive LNM (LN-prRLNM+, *N* = 129) and LN-prRLN with negative LNM (LN-prRLNM−, *N* = 249). There were no significant differences in the factors of age (*P*=0.481), multifocality (*P*=0.255), concurrent nodular goiter (*P*=0.152), and the size of cLNMa (*P*=0.630) between the two groups. On univariate analysis, sex (*P* ≤ 0.001), tumor size (*P* ≤ 0.001), extrathyroidal extension (ETE) (*P*=0.002), co-HT (*P*=0.009), cLNMa and cLNMa numbers (*P* ≤ 0.001 and *P* ≤ 0.001), and LLNM (*P* ≤ 0.001) were significantly correlated with LN-prRLN metastasis. The mean numbers of metastatic CLNs in the LN-prRLNM+ and LN-prRLNM- groups were 3 (0–29) and 0 (0–9), respectively (*P* ≤ 0.001). The mean tumor sizes were 13.9 ± 9.1 mm and 9.6 ± 6.3 mm in LN-prRLNM+ and LN-prRLNM−, respectively (*P* ≤ 0.001) (Table 3). The results of the multivariable logistic regression analysis are shown in Table 4. Following adjustment for other predictors, sex (OR = 0.61, *P*=0.116), extrathyroidal extension (OR = 1.63, *P*=0.258), concurrent Hashimoto's thyroiditis (OR = 0.62, *P*=0.104), and cLNMa number(OR = 2.03, *P*=0.051) were no longer significantly predictive of LN-prRLN metastasis. However, tumor size (OR = 1.15, *P*=0.039), cLNMa (OR = 1.28, *P*=0.001), and LLNM (OR = 1.98, *P*=0.025) were significantly predictive of LN-prRLN metastasis.

### 3.3. Prognosis

**Figure 1:**
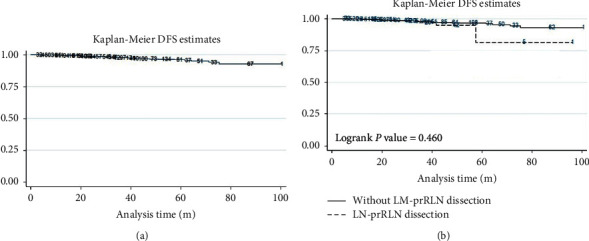
Kaplan-Meier DFS estimates in all patients (a) and the different groups (b).

**Figure 2:**
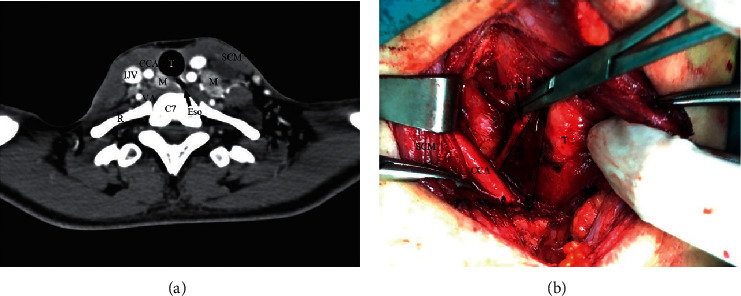
Axial contrast-enhanced CT of LN-prRLN in the cervical space. Axial contrast-enhanced CT scan shows that the LN-prRLN (M) is medial-posterior to the level of the CCA and IJV and anterior to the prevertebral fascia (aside the tracheoesophageal sulcus). B. The surgical field after LN-prRLN dissection during right-sided central compartment dissection. The part within the quadrangle with solid lines presents the detailed anatomic structure of the LN-prRLNlevel. The right CCA, IJV, and SCM were retracted laterally with a long-arm retractor. CCA: common carotid artery; IJV: interjugular vein; SCM: sternocleidomastoid muscle; T: trachea; VA: vertebral artery; C7: 7^th^ cervical vertebra; R: first rib; Eso: esophagus.

To better understand the effect of LN-prRLN dissection on recurrence, we carefully compared the postoperative complications and recurrent locationsamong the reoperation patients. The postoperative hypocalcemia and hoarseness were more in group A, but most of them were temporary, and there was no difference in permanent. It may be related to the heavier and more thorough cleaning of LN-prRLN (Table 5). The overall DFS was 97.65%, and there was no significant difference between group A and group B (*P*=0.460). The number of recurrent patients in the contralateral thyroid lobe, central compartment, and lateral compartment was 1, 2, 3 and 3, 10, 16, respectively. In particular, recurrence in the LN-prRLN region was 0 *vs*. 4 (Table 6). No significant difference was found (Figure 1), but there were still many relapses in LN-prRLN for patients who did not accept LN-prRLN dissection in the initial surgery. We have summarized the patients with relapse in the LN-prRLN region, and the specific information is shown in Table 7. In general, the recurrence of this area is not rare.  Figure 2 shows the CT and postoperative status of a patient with recurrence in the LN-prRLN region. The patient did not undergo LN-prRLN dissection in the first operation. LN-prRLN was found on the right side, and a number of enlarged lymph nodes were found in the left lateral cervical compartment during follow-up. Complete dissection was performed during the operation. The function of the recurrent laryngeal nerve was good, and there was no residual in the operation area.

## 4. Discussion

PTCs commonly metastasis to the cervical lymph nodes, which primarily occurs in the central compartment lymph nodes. The role of central lymph node dissection in preventing lymph node recurrence has been controversial, especially for patients with negative clinical lymph node metastasis (LNM) (cN0) [15]. However, recent persuasive research suggests that insufficient central lymph node dissection (CLND) after initial surgery is the common cause of recurrence in PTC cases. Therefore, a thorough dissection of the lymphatic tissue in the central compartment may reduce the risks of recurrent or persistent disease by eliminating residual subclinical LNM. Under such circumstances, attention needs to be directed toward the clinical significance of LN-prRLN, which has been frequently unrecognized during right CLND. However, given the different courses along which the RLN runs, to the left or right side, the LN-prRLN has close relationships with the right RLN, esophagus, and prevertebral fascia. Incomplete dissection of these nodes can be a cause of disease recurrence, and reoperation may increase patient morbidity and postoperative complications [16]. Nevertheless, the surgical management of lymph node LN-prRLN for PTC remains unclarified. However, the sensitivities of preoperative imaging studies, including ultrasonography and computed tomography, are not sufficient to detect LNM of the central compartment [17]. Thus, risk factors predicting LN-prRLN metastasis should be identified, as they may be valuable to evaluate the nodal status of patients with PTC before surgery and to determine whether lymph node dissection including LN-prRLN is necessary. Consequently, it is imperative to investigate the incidence and predictors of LN-prRLN in PTC.

In the present study, the incidence of cLNM was 55.6% in group A. However, the incidence of LN-prRLN metastasis was 34.13% (129 of 378 cases). In some retrospective studies, the LN-prRLN metastasis rates were approximately 11.0% to 27.2% [13, 18, 19]. Furthermore, among 1109 patients without LN-prRLN dissection in group B, 29 experienced relapse during the follow-up, of which 4 cases recurred in the location of LN-prRLN, while zero recurred in group A. Considering the rate of LN-prRLN metastasis and recurrence in this compartment, we thought that complete cLND including LN-prRLN was necessary in the initial surgery treatment.

Tumor size played an important role in stimulating LN-prRLN metastasis. Most studies confirmed that a larger tumor led to a greater likelihood of LN-prRLN metastases [13, 20, 21]. In our study, the mean tumor size in patients with and without LN-prRLN metastasis was 13.9 ± 9.1 mm *vs*. 9.6 ± 6.3 mm (*P* ≤ 0.001). In multivariate analyses, we also found that there was an association between LN-prRLN metastasis and tumor size (*P*=0.039). These results potently declared that the prevalence of LN-prRLN metastasis rapidly increases with tumor size. It has been reported that 18–87% of patients with thyroid carcinoma have multiple tumor foci [22, 23]. In keeping with this, 34.13% (129/378) of patients in our study were multifocal. Several studies have suggested that the presence of multifocality is a risk factor for cLNM in patients with PTC. However, in the present study, we did not observe a significant difference between multifocality and single tumor foci (1.29 (0.83–2.02), *P*=0.255). Since tumors with ETE have a higher degree of malignancy, they are more likely to have LNM. In this study, among 36 (9.52%) patients with ETE, the rate of LN-prRLN metastasis was 16.28%, compared with 6.02% among those without ETE (*P*=0.002). However, ETE was not identified as an independent risk factor for LN-prRLN metastasis in the multivariable analysis (1.63 (0.70–3.84), *P*=0.258). This is consistent with findings reported by Yuan et al. [9] and Bae et al. [8].

Few researchers have examined metastases with concurrent HT or concurrent benign nodular goiters. In this study, we calculated the statistics for HT and nodular goiter according to postoperative pathology. The results showed that LN-prRLN metastases were found in 36 (27.91%) patients with HT and 104 (39.08%) patients without HT. The metastatic rate was significantly higher among patients without HT (*P*=0.009). Although HT is often accompanied by enlarged lymph nodes in the central compartment region, these lymph nodes are often not malignant metastases, including LN-prRLN. Furthermore, HT provided a protective mechanism to reduce the metastatic effect. Otherwise, this phenomenon was not found in patients with nodular goiter.

The presence of right cLNM is significantly associated with LN-prRLN metastasis. Most studies have shown that right cLNM is an independent risk factor for LN-prRLN metastasis [10, 11, 13]. In our study, the proportion of LN-prRLN metastasis in the LN-prRLNM- group was substantially lower than that in the LN-prRLNM+ group (41.37% *vs* 82.95%, *P* ≤ 0.001). Multivariate analysis also confirmed that the presence of right cLNM is an independent risk factor for LN-prRLN metastasis. Furthermore, with the increasing number of metastatic right central lymph nodes, the presence of LN-prRLN metastasis also increased substantially. Furthermore, we did not find that the diameter of cLNM was associated with LN-prRLN metastasis. Similarly, a univariate analysis showed that LLNM was also significantly associated with LN-prRLN metastasis.

Our study has a limitation that should be taken into consideration. There was selection bias in these two groups. In the early stage of our center, not all patients underwent LN-prRLN dissection, but patients were young and had a larger tumor size, concurrent Hashimoto's thyroiditis or LNM. These differences were demonstrated in the comparison of group A and group B (Table 2). This retrospective study made us realize that a larger tumor size and positive LNM are real risk factors, and these groups of patients should be treated with LN-prRLN dissection. Therefore, a prospective randomized controlled trial is needed in future studies.

In conclusion, our study revealed that patients with PTC with large tumor sizes, central LN metastasis, and lateral cervical lymph node metastasis are at high risk of LN-prRLN metastasis, and these findings could assist surgeons in evaluating surgical treatment strategies. These findings provide support for the necessity of LN-prRLN dissection in such patients.

## Figures and Tables

**Table 1:** Demographic characteristics of patients enrolled in this study.

Variables	No. of patients (%) (*N* = 1487)
Age (*y*, mean ± SD)	46.14 ± 11.42
Sex (Female/Male)	1134 (76.26%)/353 (23.74%)
Multifocality (yes/No)	509 (34.23%)/978 (65.77%)
Size (mm, mean ± SD)	9.70 ± 6.73
Extensive invasion (yes/No)	129 (8.68%)/1358 (91.32%)
Co-HT (yes/No)	367 (24.68%)/1120 (75.32%)
Co-nodular goiter (yes/No)	277 (18.63%)/1210 (81.37%)
Thyroid operation methods	
RL	1016 (68.33%)
TT	471 (31.67%)
LNM	
cLNM (yes/No)	674 (45.33%)/813 (54.67%)
Positive cLNM no. (p50, (min-max))	0 (0–29)
Dissected cLNM no. (p50, (min-max))	4 (0–33)
LLNM (yes/No)	140 (9.41%)/1347 (90.59%)
Positive LLNM no. (p50, (min-max))	4 (0–37)
Dissected LLNM no. (p50, (min-max))	26.5 (0–75)
Recurrence (%)	35 (2.35%)
Follow-up (months, mean ± SD)	36.91 ± 21.43

Abbreviations: SD: standard deviation; Co-HT: concurrent Hashimoto's thyroiditis; Conodular goiter: concomitant with nodular goiter; LNM: lymph node metastasis; cLNM: central lymph node metastasis; LLNM: lateral lymph node metastasis.

**Table 2:** Demographic characteristics of patients enrolled in this study.

Variables	No. of patients (%)		*P* value
	Group A (*N* = 378)	Group B (*N* = 1109)	
Age (y, mean ± SD)	43.12 ± 11.06	47.17 ± 11.36	≤0.001
Sex (Female/Male)	291 (77.0%)/87 (23.0%)	843 (76.0%)/266 (24.0%)	0.702
Multifocality (yes/No)	129 (34.1%)/249 (65.9%)	380 (34.27%)/729 (65.73%)	0.961
Size (mm, mean ± SD)	11.14 ± 7.78	9.23 ± 6.33	≤0.001
Extensive invasion (yes/No)	36 (9.5%)/342 (90.5%)	93 (8.39%)/1016 (91.61%)	0.497
Co-HT (yes/No)	140 (37.0%)/238 (63.0%)	227 (20.47%)/882 (79.53%)	≤0.001
Conodular goiter (yes/No)	58 (15.3%)/320 (84.7%)	219 (19.75%)/890 (80.25%)	0.058
cN1a (Yes/No)	96 (25.4%)/282 (74.6%)	332 (30.0%)/777 (70.0%)	0.100
LNM			
cLNMa (yes/No)	210 (55.6%)/168 (44.4%)	464 (41.84%)/645 (58.16%)	≤0.001
cLNMa size (mm, mean ± SD)	7.86 ± 5.31	—	
Positive cLNMa no. (p50, (min-max))	1 (0–29)	0 (0–17)	≤0.001^*∗*^
Dissected cLNMa no. (p50, (min-max))	5 (0–33)	4 (0–25)	≤0.001^*∗*^
LLNM (yes/No)	88 (23.3%)/290 (76.7%)	52 (4.69%)/1057 (95.31%)	≤0.001
Positive LLNM no. (p50, (min-max))	5 (1–37)	3 (1–33)	0.022^*∗*^
Dissected LLNM no. (p50, (min-max))	27 (2–75)	24.5 (3–75)	0.089^*∗*^
LN-prRLN metastasis (yes/No)	129 (34.1%)/249 (65.9%)	—	—
Size of LN-prRLN(mm, mean ± SD)	5.82 ± 3.60	—	—
Recurrence (%)	6 (1.59%)	29 (2.61%)	0.255
Follow-up (months, mean ± SD)	23.19 ± 16.09	42.02 ± 21.12	≤0.001

SD: standard deviation; Co-HT: concurrent Hashimoto's thyroiditis; Conodular goiter: concomitant with nodular goiter; cN1a: clinical positive central lymph nodes metastasis; cLNMa:central lymph nodes anterior to the right recurrent laryngeal nerve; LNM: Lymph node metastasis; cLNM: Central lymph node metastasis; LLNM: Lateral lymph node metastasis. After the Shapiro-Wilk test and homogeneity of variance test, we found that the data did not conform to a normal distribution and that the variance was not uniform. Therefore, we used the Wilcoxon test to calculate the difference between the two groups.

**Table 3:** Univariate analysis of factors associated with LN-prRLN metastasis.

Variables	LN-prRLNM+	LN-prRLNM−	OR	*P* value
	(*N* = 129)	(*N* = 249)		
Age	42.57 ± 10.90	43.41 ± 11.15	0.99 (0.97–1.01)	0.481
Sex (female *vs.* male)	85 (65.89%)/44 (34.11%)	206 (82.73%)/43 (17.27%)	0.40 (0.25–0.66)	≤0.001
Multifocality (yes *vs.* No)	49 (37.98%)/80 (62.02%)	80 (32.13%)/169 (67.87%)	1.29 (0.83–2.02)	0.255
Size (mm)	13.9 ± 9.1	9.6 ± 6.3	2.14 (1.55–2.95)	≤0.001
Extrathyroidal extension (yes *vs.* No)	21 (16.28%)/108 (83.72%)	15 (6.02%)/234 (93.98%)	3.03 (1.51–6.11)	0.002
Co-HT (yes *vs.* No)	36 (27.91%)/93 (72.09%)	104 (41.77%)/145 (58.23%)	0.54 (0.34–0.85)	0.009
Co-nodular goiter (yes *vs.* No)	15 (11.63%)/114 (88.37%)	43 (17.27%)/206 (82.73%)	0.63 (0.34–1.18)	0.152
Central LNM				
cLNMa (yes *vs.* No)	107 (82.95%)/22 (17.05%)	103 (41.37%)/146 (58.63%)	6.89 (4.08–11.64)	≤0.001
cLNManumber	3 (0–29)	0 (0–9)	1.56 (1.39–1.75)	≤0.001
cLNMa size	0.81 ± 0.57	0.77 ± 0.51	1.14 (0.67–1.95)	0.630
Lateral LNM (yes *vs.* No)	53 (41.09%)/76 (58.91%)	35 (14.06%)/214 (85.94%)	4.26 (2.58–7.04)	≤0.001

Co-HT: concurrent Hashimoto's thyroiditis; Conodular goiter: concomitant with nodular goiter; cLNMa: central lymph nodes anterior to the right recurrent laryngeal nerve.

**Table 4:** Multivariate analysis of factors associated with LN-prRLN metastasis.

Variables	OR	*P* value
Sex (Female *vs.* male)	0.61 (0.34–1.13)	0.116
Size	1.45 (1.02–2.06)	0.039
Extensive invasion (yes *vs.* No)	1.63 (0.70–3.84)	0.258
Co-HT (yes *vs.* No)	0.62 (0.35–1.10)	0.104
cLNMa (yes *vs.* No)	1.28 (1.11–1.48)	0.001
cLNMa number	2.03 (0.99–4.13)	0.051
Lateral LNM (yes *vs.* No)	1.98 (1.09–3.62)	0.025

Co-HT: concurrent Hashimoto's thyroiditis; cLNMa: central lymph nodes anterior to the right recurrent laryngeal nerve.

**Table 5:** Comparisons of postoperative complications.

Variables	Group A	Group B	*P* value
	(*N* = 378)	(*N* = 1109)	
*Postoperative hypocalcemia*			
Temporary	28 (7.4%)	44 (4.0%)	0.012
Persistent	1 (0.3%)	3 (0.3%)	1.000

*RLN monolateral palsy*			
Temporary	12 (3.2%)	26 (2.3%)	0.352
Persistent	3 (0.8%)	15 (1.4%)	0.586
Chyle leakage	18 (4.8%)	41 (3.7%)	0.362
Hematoma	2 (0.5%)	13 (1.2%)	0.380
Wound infection	3 (0.8%)	6 (0.5%)	0.701

RLN: recurrent laryngeal nerve.

**Table 6:** Location of recurrence.

Variables	Group A	Group B	*P* value
	(*N* = 6)	(*N* = 29)	
Contralateral thyroid lobe	1 (16.67%)	3 (10.34%)	0.448
Central compartment	2 (33.33%)	10 (34.48%)	

LN-prRLN	0 (0%)	4 (13.79%)	0.515
Others	2 (33.33%)	6 (20.69%)	

Lateral compartment	3 (50.0%)	16 (55.14%)	1.000

**Table 7:** The demographic characteristics of the four patients with recurrence in LN-prRLN.

Variables	Case1	Case 2	Case3	Case4	Sum
Age (*y*)	55	62	29	22	42.0 ± 19.48
Sex (Female/Male)	F	F	F	F	4/0
Multifocality (yes/No)	Y	Y	Y	N	3/1
Size (mm)	35	12	55	15	29.25 ± 19.97
Extensive invasion (yes/No)	Y	N	N	N	1/3
Co-HT (yes/No)	N	Y	N	N	1/3
Co-nodular goiter (yes/No)	N	N	N	N	0/3
cLNMa (yes/No)	Y	Y	Y	Y	4/0
Positive/Dissected cLNMa numbers	11/12	11/21	9/15	6/7	—
LLNM (yes/No)	N	N	Y	Y	2/2
Positive/Dissected LLNM numbers	—	—	7/21	33/75	—
Follow-up (months)	31.47	33.73	39.07	23.40	31.92 ± 6.51

Co-HT: concurrent Hashimoto's thyroiditis; Conodular goiter: concomitant with nodular goiter; cLNMa: central lymph nodes anterior to the right recurrent laryngeal nerve; LLNM: lateral lymph node metastasis.

## Data Availability

The datasets used to support the findings of this study are restricted by the Ethics Committees of Fudan University Shanghai Cancer Center, in order to protect patient privacy. The data are available from the corresponding author for researchers upon reasonable request.
